# The Georgia State Dental Society

**Published:** 1885-11

**Authors:** 


AMERICAN JOURNAL

OF

DENTAL SCIENCE.

VOL. XIX. THIRD SERIES.—NOVEMBER 1885. No. 7.

ARTICLE I.

### THE GEORGIA STATE DENTAL SOCIETY.

The Seventeenth Annual Meeting of the Georgia State Dental Society convened in rooms 13 and 14, of the Pulaski House, Savannah, Georgia, May 13th, 1885.

The following officers, together with a large number of members, were present and filled their respective positions:

A. G. Bouton, of Savannah, *President.*

S. M. Roach, of Savannah, *Vice-President.*

L. D. Carpenter, of Atlanta, *Cor. Secretary.*

G. W. H. Whittaker, of Sandersville, *Rec. Secretary.*

H. A. Lowrance, of Athens, *Treasurer.*

Dr. E. Parsons, the Nestor of Dentistry in Georgia, and the oldest practitioner in Savannah, welcomed the Society in a cordial address, as follows:

MR. PRESIDENT.—The Dentists of this city have desired me to represent them in welcoming our visiting brethren who have come here to attend this the Annual Meeting of the Georgia State Dental Society. In the performance of this duty, with your permission, I will not interrupt the regular order of business but for a few minutes only.

Brethren, you have left your fields of labor at a sacrifice of both time and money, and come here to contribute, as far as you are able, to the advancing Science of Dentistry in this State. Your brother dentists in this city, through me, greet you with a hearty welcome. It will be our pleasure to make your stay here with us as pleasant as possible.

No great advance in the Arts and Sciences have been made without associated effort. By this means, the men of genius have been enabled to mature the fruit of the brains, and give to the world the marvelous improvements of the age, that which, fifty years ago, was not even dreamed of.

Without co-operation, where, to-day, would be our steamboats, railroads, telegraph, telephone, and a thousand other useful inventions which this progressive age has developed, making more conspicuous the great difference be tween the savage and civilized man. Compare the advantages of the dental student of to-day to what they were fifty years ago. Then he had no means of acquiring a proper knowledge of our profession, outside of his preceptor's office, as almost every dentist in the land had his secrets, which he held like a miser for his own advantge. Now our dental colleges, literature, and dental associations, give to the student ample means to qualify himself for the career of an intelligent gentleman and skillful dentist.

This Society had its birth in 1869, since which time it has caused laws to be enacted for the protection of both qualified dentists and the public from the malpractice of ignorant pretenders. Without legal protection our State would even now be overrun with those that have always been a disgrace to the name of dentist.

Were it not for the combined efforts of this Society you would be liable to jury duty, often interfering with your engagements, and seriously disappointing both yourselves and patients.

By associating together we have the pleasure of social intercourse, and the advantage of comparing our successes and failures with those of our brother dentists, and thereby attain to a better understanding of the *whys* and *wherefores*.

When you return home I trust you will be wiser and better dentists than when you came here, and if so, you will be doubly compensated for all that it has cost you. And now, in the name of the dentists of Savannah, I again bid you welcome--yea, thrice welcome to what little hospitality we are able to show you.

Dr. B. J. Catching, of Atlanta, Editor of the *Southern Dental Journal*, responded for the Society in a happy manner.

Dr. A. G. Bouton delivered the Annual Presidential Address, as follows:

" To tender my acknowledgements for the expression of your confidence in electing me to the position of President, is my first and most pleasing duty—an honor highly esteemed, and one I shall never forfeit.

"Another year is numbered with the past since we last met at Atlanta, and we are again assembled to exchange friendly greetings and labor for the honor and dignity of our profession. We have great reason to be thankful to a kind Providence for permitting us all to come together again. Death, the enemy who conquers all, has not, so far as I am aware, made any inroads into our membership. If any have been chastened with affliction and disease, may they look to the same divine source for sympathy and love, and be enabled to place their confidence and trust in Him who 'doeth all things well.' Thus they, too, may enter into thanksgiving with us. There is something grand and delightful in these annual reunions. We come from all parts of the State to develop and bind the social qualities that cement us together in professional sympathy and fraternal love, as well as to interchange opinions and aid one another in our deliberations upon the topics presented, and to advance the cause of dental science. Gentlemen, we, as a Society, and as individuals, have a very important work before us—one that demands the combined efforts of our best talents and energies. Let us labor to be contributors to the general fund of professional knowledge, while drawing upon it for that it has to give to each of us. Thus receiving and communicating, we shall advance the honors and dignity of the profession, and elevate the standard of our Society. Dentistry, in its scientific development, is making sure and promising progress; slow, perhaps, it may seem in this hurried and busy age, when haste and unrest is seen on every side, but it is not slow. While we think of the rapid strides made in population, in manufacture and commerce, in literature, in science and in art, as much as we may deplore many of her deficiencies, none have made greater progress than dentistry. Science cannot at once display all the ignorance, remove all prejudice, or rectify all the errors. The light comes, not in a noonday outburst of sudden and blinding light, but slowly, as the coming of the day. Let us rejoice, as we may, that the day is brightening and the future is illuminated with ever-cheering prospects. While the results of education are slow and almost imperceptible, they are nevertheless certain and momentous, with an all-pervading influence, like the light of day, the purified atmosphere, the gentle dews of heaven, imparting fertility to the earth and life and happiness to man. While upon the whole there has been great progress in dental education, we believe it should still be the leading interest of the profession. In its present influence, and in its relations to the future, it occupies no mean place in the category of the practical sciences and the useful arts. All subjects having a direct bearing upon our specialty should be perfectly familiar to every practitioner; and a knowledge of general medicine is indispensable to the thoroughly qualified dentist. The great want of our profession is a higher education of those entering its doors —men trained in habits of close and independent thought; men closely and carefully trained in mental processes, and familiar with the stored-up results of the past. With such men entering our profession, every branch would be thoroughly and carefully pursued.

The trouble is, the better class of operators are too busy to take students, and the result is, they either get with those who should be students themselves or enter College without any previous preparation, and, what is worse, with a mere desire for its diploma, and no wish or thought for its honor. His whole desire is to get his diploma as easily as possible, and go out and conquer—conquering not the mysteries that will make him master of his profession; but he argues that life is short, and that he has not time to 'labor and to wait.' He has no thought for humanity; all his thinking is done in dollars and cents.

In most cases, unless the habit of close application and study is acquired before graduation, and before the vortex of business has fastened itself upon him, the man of this uncertain education will remain destitute of any ideas outside of quick operations, bad amalgam filling, and money making.

But, gentlemen, I have said enough upon this familiar subject. I desire now to say a few words regarding the effect such an Association as this has upon the individual:

First. It wonderfully expands his affectionate nature. He who sits apart in seclusion, scorning companionship with his fellow-men, necessarily becomes unloving and unlovable, dissatisfied with himself and with his neighbor, and intensely disagreeable to live with. He never feels the glow of generous emotion; never allows himself any better pleasure than cynicism. What air is to the lungs, expanding them, are friends to man. Our hearts increase in direct ratio. The more recipients the more room. A union of hands and hearts, a union of thought such as this Society affords in the formation of life-long association; there is nothing more noble than this. Consider, also, the moral effects of such association. Man left to himself becomes morose, selfish, suspicious–finding no object of inspection outside of himself; his eyes are perpetually turned inward. When no food is furnished the stomach, its acids consume its own coating, so with this constant interspection and brooding, Not diverted by reception of fresh and varied thoughts from outside sources, it soon develops a species of insanity. As keeping the powers of the mind and heart in vigorus, healthful movement, we should especially value our Society.

Again. Communing only with himself, a man becomes bigoted, dogmatic. Unaccustomed to the interchange and comparison of ideas, all his arguments, all his convictions, furnished by self-interest, he is unprepared for the reception of other thoughts; his mind is as limited as it is rigid. No persuasion or cudgelling can introduce a single additional thought. All such thoughts as are cosmopolitan–as effect communities–as bind men together in social compact, he is utterly dead to. To prevent such debasement of our powers by mutual incitement; to lift each other to grand and magnanimous planes of thinking and feeling, and acting, our Society was formed. God has placed us here on earth, in the midst of mighty and numerous influences. Man should always save for his fellow-men the best—trash he has for himself; he should be communistic, and share all his loftier conceptions with humanity. So is the world lifted. Even if he abode in his little sanctum and conversed with books, and philosophy of life would be dangerously fallacious. Man is the proper study of mankind. The only education the majority receive is from affiliation with men. The truth in physics is true in mind. Heat expands and cold contracts. The sympathetic contact of man with man opens the mind; seclusion from him warps it. Stimulated by rivalry, like blooded horses we stretch every energy to be head and head in the race.

When we measure ourselves side by side with others after the first shock of self-conceit is over, then comes the noble aspirations to equal or excel them. There is a fire awakened in us when our views are disputed. We gather our energies for the attack and the retort. Debate is the whet-stone on which to sharpen muddy-mettled minds. Men not only accumulate power by union, but gain warmth and earnestness. The heart is kindled; an electric communication is established between those who are brought nigh and bound to one another by common labors. Union not only brings to a point forces which before existed, and which were ineffectual through separation, but by the feeling and interest which it arouses, it becomes a creative principal; calls forth new forces, and gives the mind a consciousness of power which would otherwise have been unknown. By this sympathy we may nurture a high professional honor; each one is brought under the surveilance and criticism of all the rest. By comparison of ideas, too, we take the bearings of our science; find just what each is doing; just what remains to be done. Only this is progress: otherwise we do those things we ought not to do, and leave undone those things we ought to do. Such a union of men, brought necessarily together by a common interest,in the advancement of a common profession, is a union natural, honorable and elevating. Were it not for this disposition of man to be a social being, the world would now be in barbarism. Wherever individualism reigns; wherever man but considers himself; wherever he only yields obedience to his own passions, there society becomes almost impossible. But such a state is associated with discomfort; man becomes a prey of the elements—descends to the scale of the brutes. To better himself he must join in companies, communities. Joint effort conquers nature; hews through mountains; rears pyramids. Associated with his kind, man gains dominion over the strongest animals; over the earth and sea; and, by his growing knowledge, obtains a kind of property in the universe; such is the grand principal under which we are enlisted.

I thank you, gentlemen, for your kind patience, and beg your indulgence and assistance during the session.

After this address, President Bouton called the meeting to order, and the regular order of business was taken up.

The first business transaction was the appointment of a committee to revise membership. The committee was appointed by the Chair as follows: Drs. Lowrance, Whittaker, J. P. Holmes, R. B. Adair and L. D. Carpenter.

While a recess was being taken, to allow the committee on membership to report, an impromptu clinic was held. Dr. R. B. Adair, of Gainesville, was suffering from an abscess on his left forefinger, which he wanted to have operated upon, and test the efficacy of cocoaine, as an experiment. Dr. Hopps volunteered to procure the drug, and Dr. Catching to perform the operation. An extensive application of cocoaine was used. In about ten minutes the abscess was lanced, and the patient, who submitted to the painful ordeal, expressed his gratification at the result.

The Executive Committee reported and recommended that the session be held from 10 o'clock A. M. until 2 P. M., daily, and clinic at 6 P. M., daily, with an additional session at night, if the business demanded it. They also submitted the names of the following for membership: Dr. J. D. Lanier, of Savannah; Dr. J. C. Bremer, of Blackshear, and Dr. J. D. Cone, of Ivanhoe. Dr. Catching acted as teller, and the candidates were elected.

On motion of Dr. J. L. Fogg, of Barnesville, Mr. G. E. Hughsly was admitted to a seat on the floor.

After report of the Executive Committee was disposed of, Dr. W. G. Brown read an interesting paper on "Electricity as a Motive Power Applied to Dentistry." He advocated the use of electricity as a motive power in dentistry. In order to get best results, a thorough knowledge of how to manage an electrical apparatus was absolutely necessary. An interesting discussion ensued, in which the members expressed themselves pro and con—electricity as a reliable motive power.

Dr. R. B. Adair read a valuable paper on "Riggs' Disease." The Doctor, having paid considerable attention to the study of this disease, presented some excellent ideas, which called forth a discussion from different members.

A discussion on "Capping Nearves" was entered into by Drs. Brown, Holmes, Fogg, Catching, White, Mason, Tignor, Adair, Smith, Parsons, Hopps and others. Various methods were presented and advocated.

On the subject of filling roots Drs. White, Smith, Catching, Fogg, Holmes, Mason and others gave their different methods.

Rubber in dentistry was discussed. A paper had been prepared by Dr. Tignor, of Columbus, but neglecting to bring it with him an outline of what he had written was presented.

A paper on "The Advantages of Gold as a Base for Artificial Dentures" was read, written by Dr. W. W. Ford, of Macon, Ga.

Dr. Catching, of Atlanta, read a paper on " Dental Literature," advocating a more liberal patronage of our dental literature, and a higher appreciation of the efforts of our leading authors. The dental journals should be patronized and encouraged.

Dr. Moncrief read a paper on "The Mouth as Presented in Every-day Practice—What Shall we do With It?"

Dr. E. Parsons read an essay on "Nervous Energy." It was a carefully prepared and interesting essay, which treated the subject in an original manner.

Dr.Coyle, read a highly entertaining paper on "Theory vs. Practice," embodying many excellent ideas.

Dr. Coyle brought up the subject of illegal practice in the State. He said there are a number of men engaged in practicing in Georgia in open defiance of the law.

The Doctor moved that the Executive Committee be authorized to draw upon the Treasurer for $50.00 per annum, with which to prosecute those practicing illegally. The motion was advocated by others. It seemed to be the sentiment of the delegates that the prosecuting attorneys have been too lax in the past in allowing the law to be abused. Dr Catching offered an amendment to the motion—that an individual assessment of one dollar per annum be made, if the necessity arose, aside from the appropriation. The following resolution passed:

*Resolved*, That the Chairman of the Executive Committee be authorized to draw upon the Treasurer of the Society to the amount of $50.00 during the year, if necessary, for the purpose of prosecuting persons unlawfully engaged in the practice of dentistry in the State, and that we pledge the Society, collectively and individually, to an assessment, not over $1.00 at one time, if necessary.

Dr. Parsons exhibited some plates in celluloid and rubber.

Dr. Carpenter, of Atlanta, showed some plaster models of badly deformed mouths, which he had greatly improved by treatment.

The Treasurer reported fifty one members in good standing.

The following officers were elected for the ensuing year:

President, J. H. Coyle, Thomasville, Ga.; First Vice-President, J. P. Holmes, Macon, Ga.; Second Vice-President, C. T. Osborn, Albany, Ga.; Corresponding Secretary, L. D. Carpenter, Atlanta, Ga.; Recording Secretary, W. L. Smith, Hawkinsville, Ga.; Treasurer, H. A. Lowrance, Athens, Ga.;

Executive Committee: Chairman, S. A. Bradfield, Macon; G. W. H. Whittaker, Sandersville; R. B. Adair, Gainesville; N. A. Williams, Valdosta; L. D. Carpenter, Atlanta.

Dr. W. L. Smith offered the following resolutions, which were adopted:

*Resolved*, 1st. That the heartiest thanks of the Georgia State Dental Society be tendered to Savannah dentists for their great courtesy and lavish hospitality; also, to the ladies who added so much to our great enjoyment while in their beautiful city by the sea.

*Resolved*, 2d. That to the proprietor of the Pulaski House, Mr. Jas. M. Case, thanks be extended for the royal manner in which he cared for us, and for the beautiful parlors in which our session was held.

We tender our thanks to the railroads and places for courtesies extended and accepted by us.

The Society adjourned to meet in Macon, Ga, the third Tuesday in May, 1886. During the session the dentists of Savannah invited the members to go on an excursion to Tybee, which was most highly enjoyed by all. An elegant dinner was served during the trip on the splendid steamer engaged for the excursion. Quite a number of ladies were invited to accompany the dentists, who added largely to the pleasure of the trip.

The dentists of Savannah are royal in whatever they undertake, and the Society owe them a debt of gratitude for their efforts to make the meeting a pleasant one.—*Dental Luminary*.

---

ARTICLE II.

